# Unlocking the Potential of *Artemisia scoparia*: Current Evidence and Research Gaps

**DOI:** 10.3390/biology15171466

**Published:** 2026-08-28

**Authors:** Tahani Al-Surrayai, Hanan Al-Khalaifah, Sheikh Shreaz

**Affiliations:** Food Security Program, Environment and Life Sciences Research Center, Kuwait Institute for Scientific Research, P.O. Box 24885, Kuwait City 13109, Kuwait; tsuraia@kisr.edu.kw

**Keywords:** *Artemisia scoparia*, traditional uses, phytochemistry, biological activity, arid desert ecosystems

## Abstract

The plant *Artemisia scoparia*, of the *Artemisia* genus in the Asteraceae family, is known for its broad range of medicinal applications. Recent research on the traditional applications, phytochemistry, and potential biological activity of the plant is summarized in this review. Anticancer, antioxidant, antimicrobial, anti-inflammatory, neuroprotective, and hepatoprotective properties of this plant are well known. The complex bioactive profile of the plant has been revealed by analysis of key chemical elements such as coumarins, flavonoids, phenolic acids, chromones, and essential oils. Research gaps show the necessity of toxicity analyses, clinical trials, and further bioactivity assays, to confirm the therapeutic potential of *A. scoparia*. The review also covers the morphology, distribution, traditional uses, and how this plant has adapted to desert ecosystems. This plant has the potential to be used in land restoration, herbicide, and associated plants in commercial agriculture. The present review provides an in-depth understanding of the chemical composition and promising biological activities of *A. scoparia*. These bioactivities draw attention to develop novel drugs. However, further research is needed to examine the toxicity and clinical effectiveness of this plant. Although not yet studied in poultry nutrition, *A. scoparia* shows potential as a natural feed additive based on evidence from other *Artemisia* species. Finally, the future research directions to deepen our understanding of this plant to confirm its clinical applications and use in poultry feed are also provided.

## Simple Summary

*Artemisia scoparia* is a highly adaptable desert plant with a deep history in traditional medicine, particularly for treating liver and inflammatory diseases. Its true value lies in a complex profile of bioactive compounds, including coumarins, flavonoids, phenolic acids, and essential oils, which exhibit profound hepatoprotective, anticancer, and antimicrobial effects. However, the crux of current research reveals a critical bottleneck: a severe lack of standardized toxicity evaluations and human clinical studies. Consequently, further research is needed to clarify the safety, efficacy, and clinical relevance of *A. scoparia* before its therapeutic potential can be translated into evidence-based applications. Moreover, *A. scoparia* exhibits considerable ecological adaptability, including tolerance to drought and other harsh environmental conditions, while its diverse phytochemical constituents have been associated with several biological activities. However, the potential application of these characteristics beyond traditional medicinal uses, particularly in arid-land ecological restoration, remains insufficiently investigated. Similarly, the potential use of *A. scoparia* as a poultry feed additive is currently hypothetical, as evidence from related species cannot substitute for species-specific feeding trials and safety assessments. Although previous reviews have summarized the phytochemical composition and traditional applications of *A. scoparia*, important knowledge gaps remain regarding its potential agricultural and ecological applications, as well as the systematic evaluation of its safety and clinical relevance.

## 1. Introduction

**Figure 1:**
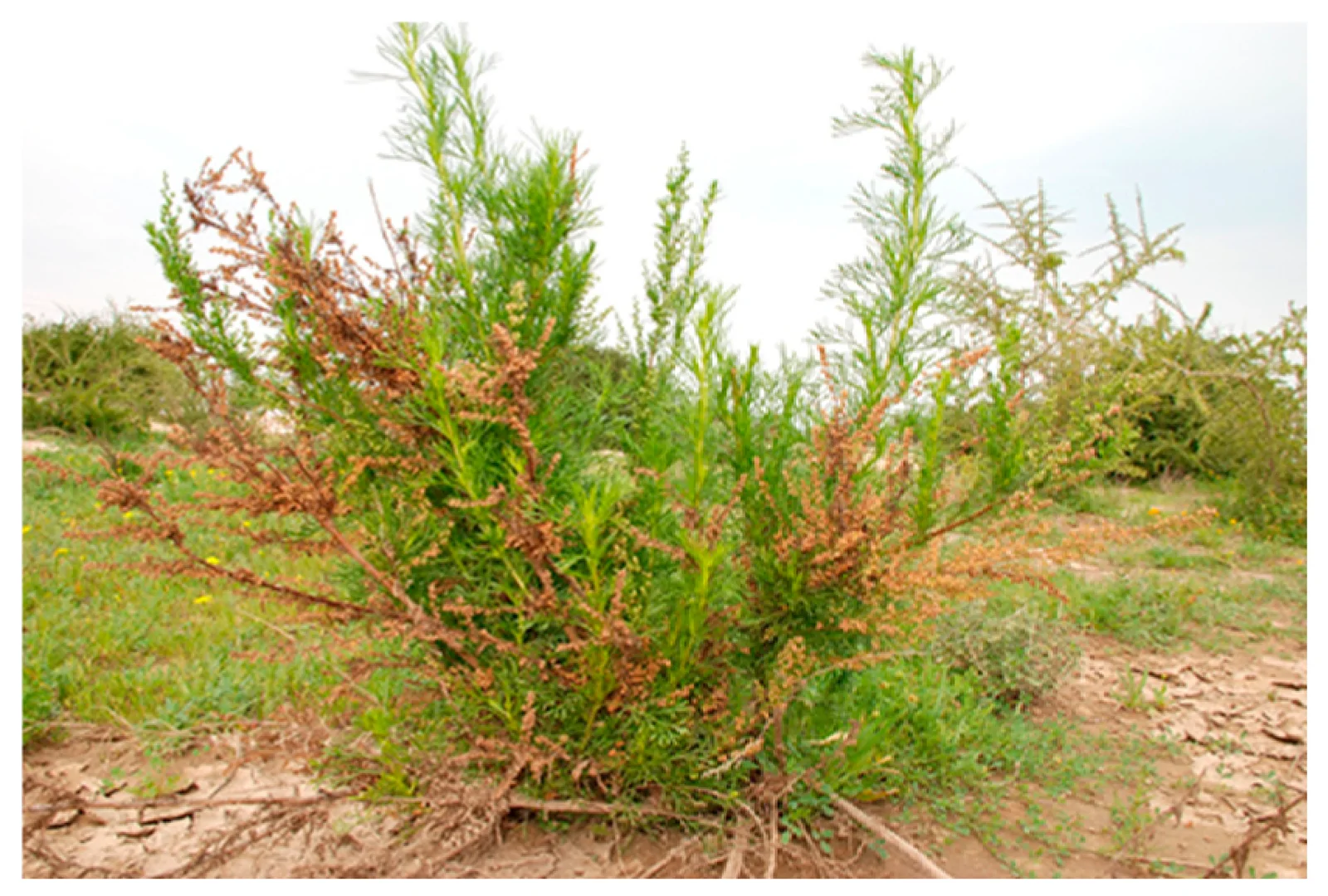
*A. scoparia* found growing wild in Kuwait deserts (Al Khiran area).

*Artemisia scoparia* Waldst. & Kit (*A. scoparia*), a resource-rich medicinal plant belonging to the Asteraceae family, has long been recognized in traditional medicine systems across various cultures for its diverse therapeutic properties [1]. However, in the context of modern pharmacology, the potential of this plant remains underexplored. As the search for effective natural therapeutic agents intensifies, *A. scoparia* (Figure 1) presents itself as a promising candidate, warranting a deeper investigation into its bioactive constituents and their potential applications [2,3,4].

Historically, this plant has been utilized for treating digestive disorders, fever, inflammation, and liver diseases [1]. Recent research provides evidence to substantiate these traditional uses, identifying key phytochemicals, including essential oils (EOs), flavonoids, coumarins, and phenolic acids, which exhibit a diverse range of biological activities [5]. Notably, the active components from *A. scoparia* have demonstrated significant pharmaceutical activities, including hepatoprotection, anticancer, antiviral, antimicrobial, antioxidant, neuroprotective as well as anti-inflammatory effects in various preclinical studies [1,3,6]. Despite these promising findings, the transition from traditional use and preliminary research to clinical application is hindered by several challenges. Among the existing approaches, the utilization of EOs from this plant has shown substantial potential [6,7,8]. However, the main limitation lies in the scarcity of toxicity studies and clinical trials. Without this crucial data, the therapeutic potential of this plant remains largely theoretical, limiting its integration into evidence-based medicine.

To the best of our knowledge, there is a deficit of comprehensive ecological surveys, targeted pharmacological investigations, and detailed phytochemical profiling regarding this desert plant specifically from Middle Eastern countries, including Kuwait. The objective of this review is to examine the research on this species, with a focus on its chemical composition and most importantly the biological activities of this plant. Additionally, we have reviewed the plant morphology, its geographical distribution, traditional uses, and how this plant adapts to arid conditions in order to provide complete and up-to-date information to carry out future research. The use of *Artemisia* species in poultry feed and its implications for *A. scoparia* are also discussed below. By identifying current research gaps and highlighting areas for future study, this study aims to deepen the insight into this plant and its therapeutic promise. While previous reviews have documented the phytochemistry and traditional uses of *A. scoparia*, an important knowledge gap remains in integrating its chemical, biological, ecological, and agricultural attributes within a multidisciplinary framework. The novelty of the present review lies in synthesizing current experimental evidence on its phytochemical constituents and biological activities with its ecological adaptability and potential sustainable applications in arid and semi-arid environments. Particular attention is given to emerging opportunities for ecological restoration and agricultural utilization, including the potential use of *A. scoparia* as a poultry feed additive. However, the latter remains a prospective application because species-specific feeding trials and safety assessments are currently lacking. This integrated perspective provides a basis for identifying knowledge gaps and defining future research priorities for the safe, evidence-based, and sustainable utilization of *A. scoparia*.

## 2. Literature Search Strategy and Selection Criteria

A structured literature search was conducted to ensure a comprehensive and transparent assessment of the available literature on *A. scoparia*. Relevant publications were identified through major scientific databases, including PubMed, Scopus, Web of Science, and Google Scholar. The search strategy employed combinations of keywords and terms related to *A. scoparia*, including “*Artemisia Scoparia*”, “*A. scoparia* essential oils”, “traditional uses of *A. scoparia*”, “phytochemistry of *A. scoparia*”, “pharmacological activities of *A. scoparia*”, “biological activity of *A. scoparia*”, “arid ecosystems”, and “poultry feed”. Additional relevant references were identified by screening the reference lists of selected publications. As this study is a narrative review, the search strategy was designed to provide broad and transparent coverage of the available evidence rather than to follow a systematic-review protocol.

### 2.1. Inclusion Criteria

Publications were considered eligible when they met one or more of the following criteria: (1) peer-reviewed original research articles investigating the chemical composition, biological or pharmacological activities, traditional uses, ecological characteristics, or potential agricultural applications of *A. scoparia*; (2) authoritative or relevant review articles providing substantive information on the phytochemistry, biological activities, traditional uses, safety, or potential applications of *A. scoparia*; (3) studies reporting verifiable experimental evidence, including in vitro, in vivo, or clinical data, where available; and (4) ethnobotanical studies documenting the traditional medicinal or therapeutic uses of *A. scoparia*. Publications were also considered according to their relevance to the objectives and scope of the present review.

### 2.2. Exclusion Criteria

The following publications were excluded: (1) duplicate records; (2) conference abstracts, editorials, opinion articles, and other non-peer-reviewed publications lacking sufficient scientific or methodological information; (3) studies without a clearly described methodological approach or adequate supporting data; and (4) publications that did not contain information relevant to the phytochemistry, biological activities, traditional uses, ecological characteristics, safety, or potential applications of *A. scoparia*. Publications for which only an English-language abstract was available were excluded unless the abstract contained sufficient information to support a specific statement made in the review.

## 3. Geographical Distribution

The genus *Artemisia* is widely distributed and comprises more than 500 species found across most of the globe with the exception of Antarctica [9,10]. *A. scoparia* is commonly found growing wild after rainfall during the summer in hot regions, particularly in sandy clay soils of arid areas, wastelands, and rural areas [11,12]. The perennial aromatic herb is notably distributed across regions such as China, Japan, Korea, Saudi Arabia, India, Central Europe, Pakistan, Iran, Kuwait and other countries [1,12,13]. This plant thrives in arid and semi-arid areas and can be found along roadsides, stony ground, rural tracks, and wastelands and is classified as a non-marine herb [1,11,12]. United States Department of Agriculture (USDA) provides a detailed distribution of *A. scoparia* across different countries within Africa, Asia (temperate and tropical areas) and Europe (middle, eastern, and south eastern) [14]. This plant is also found in desert and semi-desert environments within several areas in Saudi Arabia [1,15,16]. *A. scoparia* is also found in Kuwait, mainly in Al-Khiran area, south of the country [17]. The terrain of this area in Kuwait is a level desert plain with mild sloping hills having soils that are somewhat calcareous, non-saline, and slightly alkaline [18]. However, there are not much records published for its distribution from other Arabian Peninsula countries. There is a deficit of peer-reviewed sources for distribution of the plant as native or introduced to the particular region. The detailed list of the countries where this plant is native is provided in (Table 1), it also provides the list of the countries where this plant has been introduced [19]. More importantly, Table 1 provides an idea about the natural distribution of this plant and its expansion due to environmental factors and human activities. Perhaps most importantly, the wide geographic distribution of *A. scoparia*, extending from arid and semi-arid to temperate regions, may contribute to variation in its phytochemical composition [20]. Environmental factors such as temperature fluctuations, soil salinity and calcareous soil conditions, drought intensity, and ultraviolet (UV) radiation can influence secondary-metabolite biosynthesis and accumulation [21]. The species may therefore exhibit metabolic plasticity in response to different environmental conditions, potentially contributing to variation in the composition and concentration of bioactive constituents [22]. Such environmental influences may also contribute to the occurrence of region-specific chemotypes, particularly in EOs composition [23], as discussed in the following phytochemistry section.

## 4. Plant Morphology and Reproduction

There are large number of species in the Asteraceae family, making the genus *Artemisia* widespread genera [24]. Taxonomically, *A. scoparia* belongs to the Kingdom Plantae, Order Asterales, Family Asteraceae, Subfamily Asteroideae, Tribe Anthemideae, and Genus *Artemisia*. Morphologically, it is a biennial or perennial herbaceous plant that typically reaches 40–80 cm in height and bears either several or solitary branches in the upper part of the stem. These branches may or may not be hairy. The stems, which arise from a branched rootstock, are generally purplish brown in colour. Initially, the stem branches and leaves are sea green; later, they become yellowish as the plant matures. The plant features 10–12 yellowish florets, with 5–6 marginal florets, all of which are fertile. These florets are approximately 0.7 mm long, tubular in shape, and have a 2-dentate corolla [25]. The leaf blade is 2 to 3 pinnatisect, elliptical to ovate-oblong, and ranges in length from 1.5 to 5 cm. There are 3–4 pairs of leaf segments and 1–2 pairs of lobules in each pair. Sessile, 1.5–2 cm long, oblong to ovate-oblong, and 2 pinnatisect with two-three pairs of segments are the characteristics of the middle cauline leaves. The leaf lobules are filiform, often curved, and 4–8 mm long, and the topmost leaves are linear or oblanceolate [11]. There are five to ten disc florets and five to seven ray florets. The shape of the cypselas is obovoid or oblong, and the ovary is retreating and is reported to be sterile.

Reproductive biology of this plant is not largely studied. It exhibits optimal reproductive traits in calcareous soil, where soil water-soluble carbon content and moisture are key factors, significantly impacting the plant’s inflorescence mass, number, and quality in the desert habitats [26]. Rapid growth, blooming, and seed generation are all part of this lifecycle strategy, which enables the plant to swiftly acquire accessible environments. The plant produces small, wind-pollinated flowers arranged in heterogamous capitula and reproduces both sexually and vegetatively. This arrangement enhances genetic diversity and adaptability in arid environments by permitting both self-pollination and cross-pollination through the presence of exterior female and central hermaphrodite florets [27]. Furthermore, this desert plant spreads by rhizomes, which allows it to successfully colonize and stabilize the dry soil of the arid environments. A thick root stock is formed which is rhizomatous and sprouts, this continues and the rhizomes keep increasing year after year [28].

## 5. Adaptations for Arid Environments

The adaptation of this plant to arid climate and particularly in the desert environment is the result of genetic evolution. These include morphological adaptations in plant or specialized proteins helping in draught and temperature tolerance. *A. scoparia* has evolved for specialized foliar anatomical characters adapted for arid environments [29]. The leaves of this plant have ring palisade, water storage parenchyma evolved from spongy tissue and secretory cavity which makes it specially adapted to arid regions [30]. Wang et al. examined that out of several species of *Artemisia*, *A. scoparia* showed characters like dense trichomes, which makes it resistant to heat and prevent excessive water loss. Alongside, thicker palisade tissues, thicker blade, leaf tightness characters also influence the temperature tolerance [31]. A fascinating ability of *A. scoparia* (C3 plant) is to exhibit ability of vascular tissues to act like Crassulacean acid metabolism (CAM) plants. The characters of leaves are reported to vary with the elevation, which is relative to the change in water availability in sunny hilly slopes, hilltops or shady hilly slopes. The plant shows low relative water content, lesser chlorophyll but higher antioxidant activity to cope with the biotic stress [30]. The optimal soil conditions for growth of this plant include sandy, loamy, and well-drained soils, which also include middle eastern hot and sandy conditions. It can grow in soils with acidic, basic, or neutral pH and is capable of enduring drought conditions. Its flowering period spans from July to August in China [1,32], while as in Middle East countries including Kuwait the flowering period spans from January to March.

The draught adaptation in this plant is not well understood, but its close relative *A. annua* has been highly researched for draught tolerance. Abscisic acid mediated *AaABCG40* gene expression in roots, leaves and buds was found to be responsible for drought resistance in *A. annua* [33]. In *A. scoparia*, moisture limiting condition shows rise in peroxidase activity, ascorbate and soluble sugar content [30]. On other hand Liu et al. [34] reported that the genes responsible for the metabolism of ethylene, indole acetic acid, and abscisic acid interact with a variety of transcriptional regulatory factors, such as receptor kinases, members of the fundamental family helix-loop-helix, APETALA2/ethylene-responsive element binding protein family, v-myb avian myeloblastosis viral oncogene homolog, and some heat shock proteins to improve the performance for drought resistance in *Artemisia* species. There is a limited research on the disease and pest response on *A. scoparia*, but Zhang et al. [35] grafted chrysanthemum on *A. scoparia* and found differential gene expression of several genes in chrysanthemum, improving the aphid resistance. There might probably be innate pest resistance in the plant, which make it survive in the wild. The salt stress in this plant is regulated by the soluble carbohydrates, proline and potassium ion gradient distribution across phyllospheric and rhizospheric parts, which keeps an osmotic balance and helps this plant to survive in the saline and arid environments [36]. Restoring ecological functions and conserving biodiversity in a desert environment can be achieved in large part by understanding the physiological responses and needs of native desert plants [37].

As per the available reports there has been a limited research on how *A. scoparia* adapts to the arid environments in the Kuwait deserts, however some available reports on other native desert plants species from the neighboring countries suggest that restoring ecological functions and conserving biodiversity in the desert ecosystems can be achieved in large part by understanding the physiological responses to abiotic factors and the interaction of plant microbiome with soil arbuscular fungal microbiota particularly in extreme arid desert environments [38,39].

## 6. Traditional Uses

**Figure 2:**
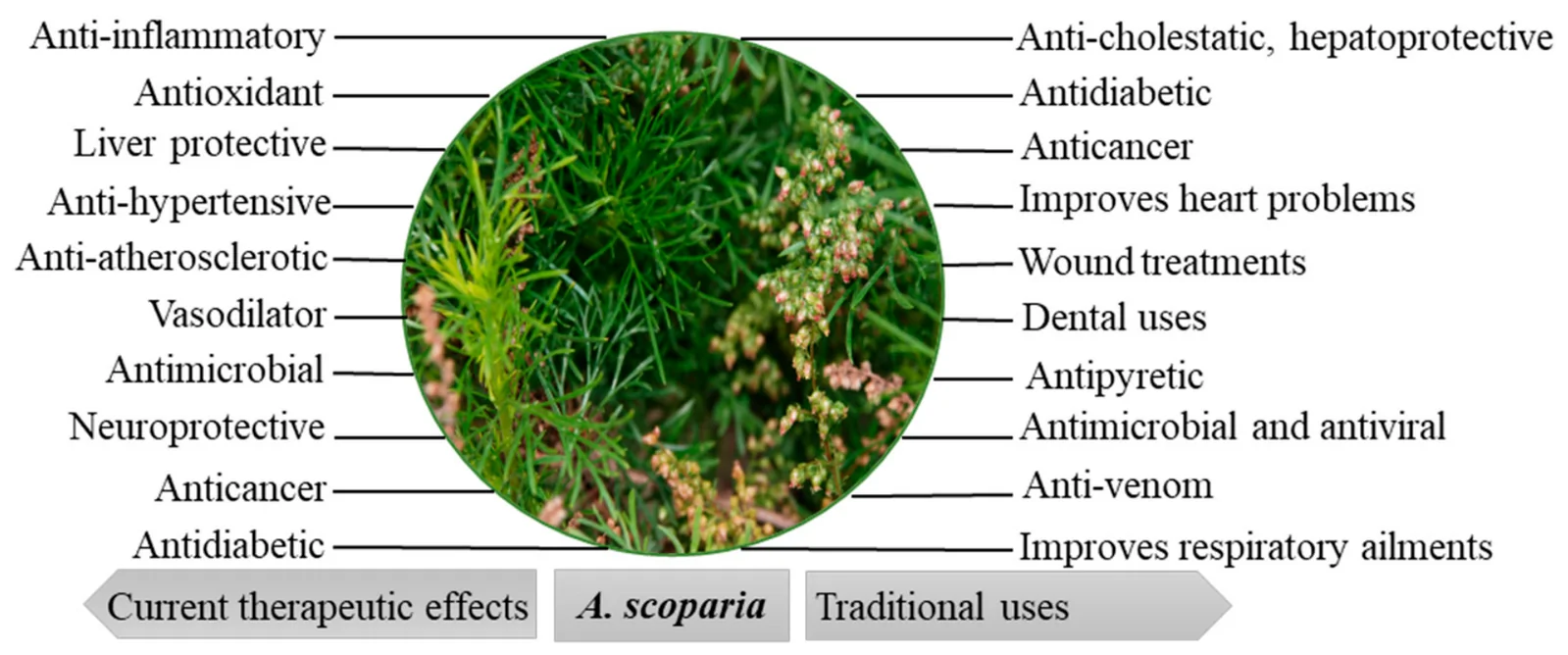
Traditional uses and current therapeutic effects of *A. scoparia* and its important constituents [7,68,69,70,71].

Current pharmacological research has shown that *A. scoparia* possesses a variety of therapeutic potentials, including anti-inflammatory, anticancer effects [7,40], antiviral [41], antiobesity, antidiabetic, antioxidative, antimicrobial [42], analgesic, and hepatoprotective activities [1]. Traditionally, as an anthelminthic agent, the leaves from this plant that have been dried and crushed are usually taken orally with water. Moreover, seasonal fever is treated with a decoction of its flower buds, especially in the summer season [43]. In Pakistan’s Gujranwala area, the plant leaves are commonly used as an antidote. Leaf extracts are used to prevent graying of the hair and strengthen it; the extracts are made by boiling leaves in coconut oil. The entire plant is pulverized and used as a purgative, and an infusion of the shoots and flowers is consumed orally to cure malaria [44]. In Khyber Pakhtunkhwa province, the shoots of the plant are taken orally to reduce fever, purify the blood, and treat liver and kidney ailments [45]. Parveen et al. reported the use of this plant to cure earaches and burns [46]. Furthermore, *A. scoparia* is utilized as an aphrodisiac in India [47]. This wild herb’s roots are used to treat hepatitis, diabetes, and cancer in the Koh-e-Safaid Range, which is located near the northern borders of Pakistan and Afghanistan [48]. *A. scoparia*, often referred to as Baeiteran/Aweejan has traditionally been used to treat liver disorders and jaundice in Saudi Arabian traditional medicine [49]. *A. scoparia*, often known as Joroob in Tajikistan grows in steppes, low mountain zones, river valleys, and deserts in Tajikistan, where this plant is used to treat hepatitis, malaria, and some other infectious diseases caused by bacteria, helminths, viruses, and fungi [50]. This plant has a lengthy history of its use in medicine across Asia, with several nations having their own unique and common cures [48,51]. Table 2 lists the cultural and historical importance of *A. scoparia* in traditional medicine in different nations and areas [1,11,44,48,49,51,52,53,54,55,56,57,58,59,60,61,62,63,64,65,66,67]. Figure 2 also highlights the wide range of ailments and diseases for which the plant has been used, offering a glimpse into its diverse traditional uses and modern medicinal applications [7,68,69,70,71].

Because of the choleretic and hepatoprotective properties, this plant is highly valued in Traditional Chinese Medicine (TCM). Its applications are recognized in the present Chinese Pharmacopoeia [72] and thoroughly detailed in the canon of TCM literature [66,73]. The dried aerial parts of *A. scoparia* or it’s another close relative, *Artemisia capillaris*, are known as *Artemisia Scopariae* herba (ASH) or Yin chen, a promising herbal medicine for treating liver diseases [74]. As per the available reports, the aerial parts from this plant are used to make TCM medicines, the people from Pakistan mostly use the roots of this plant, and in Iran, flowers from this plant are reported to be used in the local traditional medicine.

## 7. Important Plant Phytochemicals

**Figure 3:**
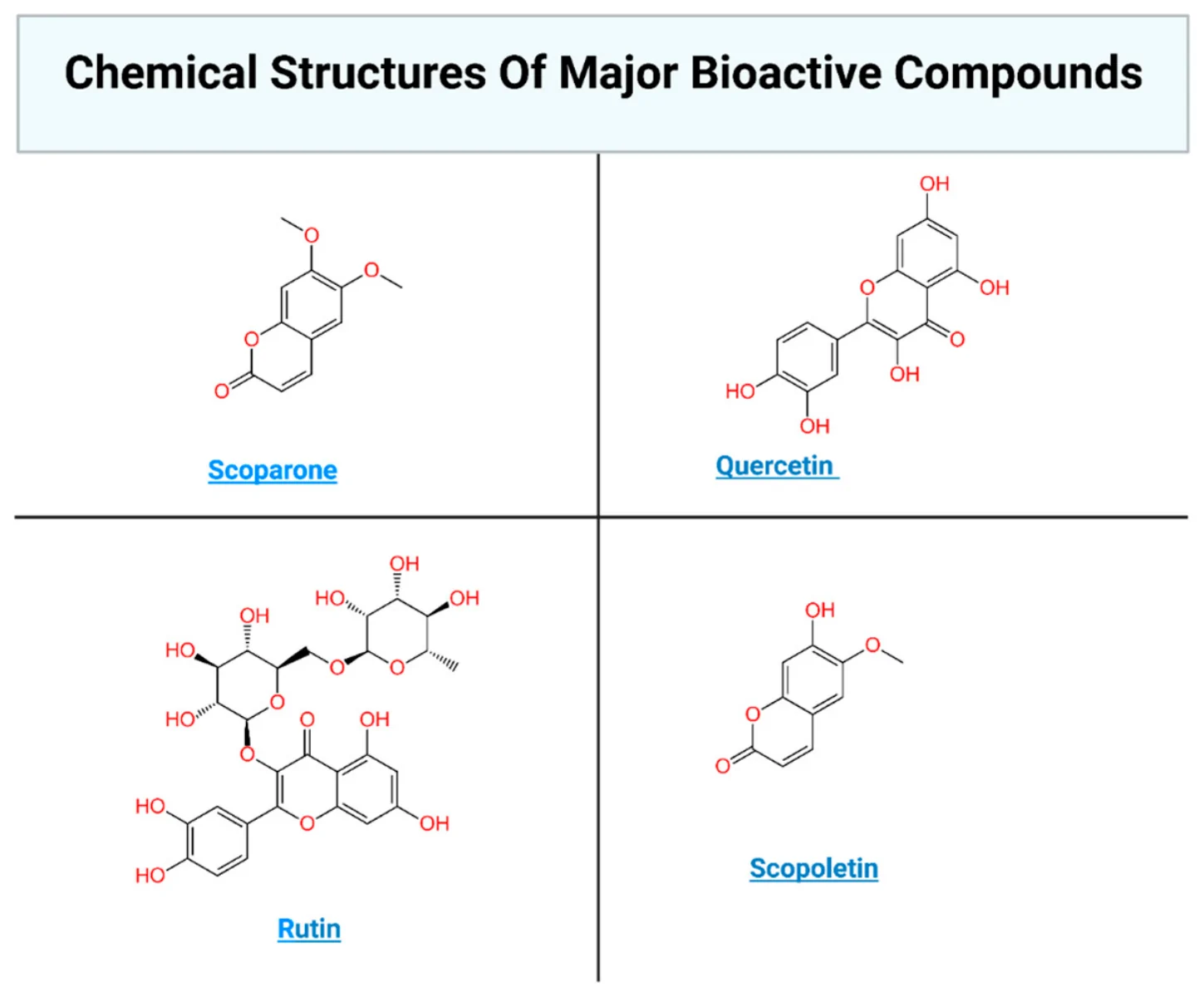
Chemical structures of major bioactive compounds.

The plant contains a diverse and complex array of chemical constituents [4]. To date, more than 100 bioactive compounds have been identified from *A. scoparia*, including flavonoids, coumarins, phenolic acids, chromones, terpenoids, glycosides, and steroids [1]. Among the important phytochemicals reported in *A. scoparia*, scoparone, scopoletin, quercetin, and rutin are notable bioactive constituents representing the coumarin and flavonoid classes. Their chemical structures are presented in (Figure 3). As per the available reports, coumarins are key bioactive components isolated from *A. scoparia*, including five simple coumarins, four furanocoumarins, one isocoumarin, and two coumarin glycosides [1]. Coumarin derivatives, scoparone, scopoletin, and esculetin isolated from this plant have been extensively studied for their pharmacological effects. Long recognized for its hypotensive and vasodilatory effects [75,76], scoparone is a common ingredient in TCM preparations. It has also been shown to have a range of other biological effects, including cardiovascular [77], hepatoprotective [78], anti-inflammatory [79], antioxidant [80], immunosuppressive [81], and antimicrobial activity [82]. Another coumarin derivative, scopoletin, found in *A. scoparia*, is a fluorescent compound by nature and is found in many plants. For many years, it has been used for sensitive hydrogen peroxide detection tests [83]. Multiple studies have shown the antioxidant [84], anti-inflammatory [85], antidepressant [86], anticonvulsant [87], neuroprotective [71], antidiabetic [88], anticancer [89], antitussive [90], osteoprotective [91], gastrokinetic [92], and antimicrobial [93] properties of scopoletin. Similar to scoparone and scopoletin, esculetin is one more coumarin derivative from the same plant, its well-established anti-inflammatory and antioxidant capabilities [94] are reported to have multiple beneficial effects within the biological systems.

Flavonoids and chromones are the another important class of compounds found in *A. scoparia*. Flavonoids are present in larger quantities in most of the plants, these compounds have a wide range of effects across multiple biological systems and have been well investigated for their unique bioactivities. Although *A. scoparia* contains a large number of flavonoids, the relative quantity of these compounds varies greatly between plants and extracts from various sources [60]. In one study, Ranjbar et al., using an aluminium chloride colorimetric technique, showed that the most abundant components in *A. scoparia* ethanolic extracts, flavonoids, were measured to be 61.1 ± 0.4 μg QE/mg [95]. Cirsmaritin, an important flavonoid from this plant has been reported to have anti-inflammatory, antimicrobial, antioxidant [2], anti-cancer [96], and antidiabetic activities [97]. Other important flavonoids reported within this plant include quercetin, isorhamnetin, rutin, naringenin, and blumeatin, all of these compounds are reported to have potential cardiovascular, hepatoprotective, antimicrobial, anti-inflammatory and anticancer effects [2]. Chromone capillarisin is one more important compound present within this plant. Although this compound is not known to be a bioactive component of other plants. Capillarisin has been reported to promote penile erection in rabbits [98], and is also shown to have anti-inflammatory, antioxidant [99], anti-asthmatic [100] and anticancer properties [101].

Among the phenolic acids, chlorogenic acid and some of its derivatives are abundant in *A. scoparia* and have been reported to show significant biological effects [102]. Although prenylated coumarin acids, a different class of compounds, are not thought to be major components of this plant, some of them, such as drupanin and capillartemisin B, have been found in this plant [103]. It has been observed that certain prenylated coumarin acids, such as cis-scopa-cis-coumaricin and cis-scopa-transcoumaricin and others found in *A. scoparia* exhibit strong adipogenic properties [104].

This plant is also high in organic acids such as phenylpropionic acids, caffeic acids, and simple phenolic acids, with chlorogenic acid being a key quality control marker for young seedlings. Several caffeoylquinic acids present within the aerial parts exhibit notable anti-inflammatory activities. Unique alkyne compounds from this plant, like 3S,8S-dihydroxydec-9-en-4,6-yne1-O-(6-O-caffeoyl)-β-D-glucopyranoside, shows strong activity against hepatitis B virus [105]. This plant also contains high levels of sesquiterpenes and is a source of artemisinin, an antimalarial drug, with a yield of about 0.015% in the aerial parts [106]. There are reports that show the presence of some other important types of compounds that include aliphatic acids, triterpenes, fatty alcohol, and sterols or its derivatives in *A. scoparia*. This plant shows the presence of *p*-hydroxyacephenone, a compound with remarkable cholagogic ingredient, notably, this compound is not found in the other closely related species of genus *Artemisia*. Significant amounts of unsaturated fatty acids and saturated fatty acids were found in the fatty acid composition of this plant in one investigation. Major saturated fatty acids include palmitic acid (20.39%) and stearic acid (11.76%). The total presence of eight saturated fatty acids is 58.01%. Key monounsaturated fatty acids are palmitoleic acid (4.53%) and oleic acid (6.23%), with a total of 10.76%. Prominent polyunsaturated fatty acids are linoleic acid (14.42%) and arachidonic acid (3.17%), totalling 31.3%. The overall unsaturated fatty acids content is 42.06% [1,107]. The major vitamin and sterol reported within this plant is retinol acetate (1.71 µg/g) and stigmasterol (2.11 µg/g) [108]. The same authors also report the significant composition of flavonoids in *A. scoparia*. However, many reports show different composition of flavonoids in different *Artemisia* species [109]. Also, there are some reports that show low or no flavonoids found in some *Artemisia* species [110].

## 8. Essential Oils (EOs)

Most plant species within the *Artemisia* genus are aromatic and produce EOs, which are commonly utilized in both perfumery and in medicine as anti-infective [13]. Till now more than 100 volatile oil constituents have been reported from *A. scoparia* through GC-MS analysis [7,106]. Notable bioactive compounds found in the EO of this plant, include β- and α-thujone, camphor, 1,8-cineole, and camphene, the presence of these compounds contribute to the scent and medicinal activities of the oil. β-thujone and α-thujone are two isomeric compounds commonly found in *Artemisia* species [13]. They belong to the class of monoterpenes and possess a unique bicyclic structure. These metabolites have been studied for their significant bioactivities, including antimicrobial, antiviral and insecticidal properties. It has been proven that EOs from *A. scoparia* contain antioxidant and free radical-scavenging qualities [6], which may be related to enhanced metabolic function. The different oil compositions found in the same plant from different regions show that the same plant can have different oil dependent chemotypes [111]. Each location has a different mass proportion of EO extracted from the upper aerial portion of this plant. That varies from 0.29% to 0.79% in Turkmenistan. In Uzbekistan, plant leaves held 1.28% EO, while their stems and inflorescences held 0.22%. The wild *A. scoparia* in Kazakhstan has an EO concentration varying from 0.08% to 1%. The EO concentration of wild plants in the Caucasus ranged from 0.4% to 1.5%. It ranged from 0.1% to 0.8% in Kalmykia [112]. However, it has been noted that the majority of the EO from Tajikistan is made up of diacetylenes, with 34.2% of the oil being 1-phenyl-2,4-pentadiyne [50]. The EO from the South Korea is reported to be high in beta-caryophyllene (6.8%), camphor (11.0%), and 1,8-cineole (21.5%) [7]. Limonene (0.1–6.3%), α-pinene (0.2–10.1%), p-cymene (0.6–15.2%), germacrene D (11.5–40.3%), β-pinene (0.4–8.9%), caryophyllene oxide (4.3–15.6%), caryophyllene (4.6–13.8%), spathulenol (4.0–11.7%), and trans-β-ocimene (0.3–5.4%) were the principal chemical constituents extracted from the EO of *A. scoparia* obtained from Buryatia and Mongolia [23]. The major component of the EO from both India and Kazakhstan was found to be p-cymene. In the EO of this plant from India, other main components included limonene (12.53%), and β-myrcene (13.95%) [113]. In contrast, the oil obtained from this plant in Kazakhstan featured methyl eugenol (27.5%) and spathulenol (8.76%) alongside β-pinene (13.6 %), and β-ocimene (10.3 %) [114]. Acenaphthene, curcumene, and (+) caryophyllene oxide were the main constituents of the EO in the plants in Northeast China [90]. Through the process of hydrodistillation, the EO from recently harvested leaves that were growing in wastelands near Chandigarh in India was extracted. The oil from young leaves included 44 different compounds, whereas the oil from mature leaves contained 31 constituents, according to the GC/MS data, which showed a composition that was primarily monoterpenoid (64–67%). The predominant components in the immature leaves were β-myrcene (24.13%) and in the mature leaves, p-cymene (27.06%) [115]. The same plants cultivated in Saudi Arabia produced EOs that were high in 2-nonanone (55%) and 2-undecanone (24.5%), which exhibited potential antimicrobial activity against six strains of bacteria and three strains of fungi [116]. However, the EO extracted from the same species in the northern region of Saudi Arabia predominantly composed of acenaphthene, accounting for 83.23% [15]. The EO yield of Iran was 0.7%, with major constituents including methyl eugenol (18.53%), spathulenol (4.37%), caryophyllene oxide (5.69%), and sabinene (1.24%) indicating that the composition of the EO varied under different ecological conditions [117]. The major constituents found in the EOs of this plant are mainly eugenol, camphor, p-cymene and limonene across different regions, still there remains further exploration of new constituents from EOs that have efficient potential in medicinal, pharmacological and agricultural areas. While extensive studies have analyzed the EO constituents of other *Artemisia* species from Middle Eastern countries [118,119,120], research on *A. scoparia* from these regions, more importantly from Kuwait remains limited and requires further exploration.

## 9. Biological Potential

To provide a balanced and critical interpretation, the following evaluation distinguishes among the different levels of scientific evidence supporting the reported biological and therapeutic activities of *A. scoparia*, including in vitro cell-based assays, in vivo animal models, and limited human observations. The reported activities should be considered in relation to the plant part examined, the bioactive constituents identified, and their proposed mechanisms of action [1]. Several important bioactive constituents, including the coumarins scoparone and scopoletin and the flavonoids quercetin and rutin, have been reported in the aerial parts of the plant, including the leaves, stems, and flowers [60]. Experimental studies have provided evidence that these compounds can modulate specific molecular pathways associated with various biological activities. For example, scoparone obtained from the aerial parts of *A. scoparia* has been reported to exert anticancer effects in prostate cancer models by inhibiting the phosphorylation and accumulation of signal transducer and activator of transcription 3 (STAT3) [121]. Scoparone has also demonstrated anti-inflammatory activity, partly through inhibition of the Toll-like receptor 4 (TLR4)/nuclear factor kappa B (NF-κB) signaling pathway [78]. Similarly, 3,5-dicaffeoyl-epi-quinic acid, a phenolic compound identified in *A. scoparia* fractions, has been reported to exhibit anti-inflammatory activity by suppressing caspase-1 activation and inhibiting NF-κB nuclear translocation [101]. The antimicrobial activity reported for *A. scoparia* essential oils has been associated with disruption of microbial cell membranes, leading to membrane damage and cellular leakage; essential oils are predominantly synthesized in the leaves and flowers [116]. Collectively, these findings provide experimental support for associations between specific bioactive constituents, their proposed molecular mechanisms, and the biological activities reported for *A. scoparia*. Nevertheless, most of the available evidence remains preclinical, and further well-designed in vivo studies and clinical investigations are required to establish the safety, efficacy, and therapeutic relevance of these findings.

*A. scoparia* has a wide range of biological activities which has gained considerable attention from researchers globally. The evidences of some important phytochemicals like flavonoids, coumarins, phenolic acids, and EOs, within this plant is well recognized across Asia and Europe. The literature provides enough evidence of its anti-inflammatory, anticancer, neuroprotective, antidiabetic, antimicrobial, and antioxidant activities. Furthermore, this plant is a strong contender for usage in liver health-related disorders due to its significant hepatoprotective properties. Therapeutic potential of this plant is due the diverse chemical composition to affect a wide range of biological processes, which positions it as a possible natural treatment for use in both traditional and modern medicine. Table 3 provides the list of different chemical constituents obtained from *A. scoparia* responsible for various biological activities [1,6,7,12,35,40,42,60,68,69,70,71,78,81,88,102,103,105,106,116,121,122,123,124,125,126,127,128,129,130,131,132,133,134,135,136,137,138,139,140,141,142,143,144,145,146,147,148,149,150,151,152,153,154,155,156,157,158,159,160,161,162,163,164,165,166,167,168,169,170,171,172,173,174,175,176,177,178,179,180,181,182,183,184,185,186,187,188,189,190,191,192,193,194,195,196,197,198,199,200,201,202,203,204,205]. This plant can be a good candidate crop for metabolite extraction if not grown in heavy metal contamination [206]. The biological potential of this plant and its important metabolites is described below in detail.

### 9.1. Antimicrobial Properties

The presence of EOs and bioactive chemicals in *A. scoparia* is responsible for its significant antibacterial activities [1]. Research has revealed that EOs of this plant have the ability to suppress a wide range of microbes, including fungus, bacteria, and parasites. A study by Cha et al. reported that the EOs derived from this plant show significant antibacterial activity when treated against different oral bacteria evaluated in their investigation. Their research examined the effect of EOs from this plant against a group of bacteria that included four facultative anaerobic bacteria (MICs 0.2–0.8 mg/mL), and three obligate anaerobic bacteria (MICs 0.025–0.2 mg/mL) [7]. *A. scoparia* EOs include a range of volatile aromatic chemicals that contribute to its antibacterial activities, including camphor, 1,8-cineole, β-thujone, and α-thujone as the main compounds [32]. These metabolites can operate against microbes in a variety of ways, more frequently these aromatic chemicals react with the lipid part of the bacterial cell membrane and cause rupturing of the cell or the mitochondrial membranes, that leads to the cell leakage and ultimately causing the cell death [116]. More importantly, the EOs from this plant contain thujone that have been reported to possess significant antimicrobial properties against the different strains of bacteria and fungi [138,207]. Furthermore, both α- and β-thujones from this plant are already been reported to show the antimicrobial effect [138,139], and when the two isomers were tested in a mixture, a synergistic interaction between the two thujone isomers was also observed [208]. Similarly, 1,8-cineole found within the same plant has demonstrated antimicrobial effects against a wide range of bacterial and fungal strains [140]. 

Using the minimum inhibitory concentration (MIC) and agar diffusion methods, Ramezani et al reported the antimicrobial activity of methanol extract of *A. scoparia* against *Bacillus subtilis*, *Staphylococcus aureus* and *Candida albicans* ATCC 10231 [141]. Ethyl acetate, diethyl ether, and hexane extracts of *A. scoparia* were tested against different bacterial and fungal strains like *B. subtilis*, *C. albicans*, *S. aureus*, *Salmonella abony*, and *Escherichia coli*. All the three extracts were found to be ineffective against gram negative bacteria but efficient against yeast and gram positive bacteria [142]. The EO of this plant identified 23 chemical compounds with tocopherol derivatives being the most abundant. This EO demonstrated strong antibacterial effects against *E. coli* and *Shigella flexneri* strains with 55–70% and 60–75% inhibition [126]. In another study, the EO from this plant is reported to be active against three Gram-positive bacteria, three Gram-negative bacteria, and three fungal strains. The EO is reported to show the highest activity against *Enterobacter xiangfangensis*, *S. aureus*, and *C. albicans* [116]. Similarly, Khan et al, and Farzaneh et al. also confirmed the potential antimicrobial activity of *A. scoparia* [68,127]. While many studies on the antimicrobial efficacy of this plant have been studied against human pathogenic bacteria and fungal strains, but there remains a knowledge gap on the activity of this plant against plant pathogenic microbes.

### 9.2. Anticancer Activities

The anticancer activities of *A. scoparia* and its bioactive compounds have been the focus of extensive research. In one study, the biosynthesized zinc oxide nanoparticles using *A. scoparia* extract showed significant activity against Huh-7 liver cancer cells. Both the plant extract and synthesized nanoparticles caused apoptosis and decreased cell proliferation with an IC_50_ value of 310.24 µg/mL and 10.26 µg/mL [128]. A key component of the molecular basis of breast cancer classification is estrogen receptor-a (ER-a). The anti-proliferative properties of this plant were evaluated using the ER-a positive and ER-a negative breast cancer cell lines, T47D and HS578T. At doses of 100–200 mg/mL, the extract considerably suppressed the proliferation of T47D cells, but had less of an impact on HS578T cells [69]. In the same investigation, the extract from this plant was reported to increase the number of apoptotic cells by 55.4%, compared to the controls used in their experiment. With an IC_50_ value of 41.3 μmol/L, scoparone, a coumarin derived from *A. scoparia*, suppresses signal transducer and activator of transcription 3 (STAT3) activity and proliferation in DU145 prostate cancer cells. The reduction of STAT3 phosphorylation and nuclear accumulation leads to the suppression of transcription of the STAT3 target gene. Additionally, DU145 xenograft tumor development was inhibited in nude mice treated for 18 days with scoparone (30 mg/kg), which was correlated with lower STAT3 phosphorylation. These findings suggest that the antitumor effect of scoparone against the prostate cancer cells is via the STAT3 inhibition [121]. A notable coumarin obtained from the same plant called scopoletin has also shown promise as an anti-tumor agent [130]. Silver nanoparticles synthesized using *A. scoparia* extract are reported to show dose-dependent toxicity against A549 lung cancer cell line. Additionally, the gene expression analysis showed up-regulation of Bax gene and down-regulation of Bcl2 gene [133].

In Huh-7 hepatocarcinoma cells, the chitosan and myristic acid nanogel-encapsulated *A. scoparia* extract demonstrated more cytotoxicity than the free extract; it induced apoptosis by up-regulating of the Bax and miR192 genes and suppressing the Bcl-2, MMP2, MMP9, and cyclin D1 genes [134]. Another study investigated the mechanism of cell death induced by the plant extract in rhabdomyosarcoma cells. Optimal uptake of the extract occurred within 1 h of incubation. The plant extract at 150 µg/mL concentration killed over 70–80% of rhabdomyosarcoma cells. This study showed that methanolic *A. scoparia* extracts were particularly effective, killing 88–93% of cancer cells compared to 23% by the chemo-drug after 48 hours, indicating significant anticancer activity [129]. Similarly, Khan et al, reported that at an IC_50_ value of 6.93 µg/mL, the ethanolic extract of the plant showed potential anticancer activity against the THP1 human monocytic leukemia cell line [68].

### 9.3. Anti-Inflammatory and Antioxidant Effects

*A. scoparia* is reported to show significant anti-inflammatory activities, such as preventing heat-induced protein denaturation in vitro [68,209] and lowering the production of inflammatory cytokines, cell infiltration, and edema in rats and mice with acute inflammation induced by carrageenan [12]. Similarly, in a mouse model of atopic dermatitis, applying the plant extract topically reduced circulating histamine levels, inflammatory cytokine levels, cell infiltration, clinical symptoms, and caspase-1 activity in lesions [148]. The anti-inflammatory effects of different *Artemesia* species can be attributed to the presence of bioactive compounds, particularly phenolic acids and flavonoids [210]. Many inflammatory mediators and enzymes involved in the inflammatory response have been shown to be inhibited by these plant chemical constituents. By targeting these inflammatory pathways, the *Artemesia* species may offer potential therapeutic benefits in the management of inflammatory disorders. Flavonoids with anti-inflammatory effects include kaempferol and quercetin. These bioactive constituents have the ability to suppress the inflammatory response by interfering with the synthesis and function of pro-inflammatory mediators such as prostaglandins and cytokines [40]. Quercetin is also reported to possesses multiple biological activities including potential anticancer activity [211]. Phenolic acids like chlorogenic acid and caffeic acid also play a vital role in the anti-inflammatory potential of *A. scoparia* [1]. These compounds have the ability to block the actions of enzymes like lipoxygenase and cyclooxygenase, which play a part in the production of mediators that cause inflammation. By blocking these enzyme functions, phenolic acids can help regulate the inflammatory response [146]. The anti-inflammatory properties of *A. scoparia* and its active compound 3,5-dicaffeoyl-epi-quinic acid (DEQA) on human mast cells were investigated. Both the plant and DEQA significantly reduced inflammatory markers (TSLP, TNF-α, IL-1β, and IL-6) by inhibiting caspase-1 activity and blocking NF-κB nuclear translocation. Additionally, the plant decreased phosphorylated-JNK levels, while DEQA also reduced phosphorylated-p38 MAPK. These results suggested that this plant could treat mast cell-mediated inflammatory diseases [101]. Hexane and dichloromethane fractions of the same plant inhibited pro-inflammatory factors in RAW264.7 murine macrophage cells. Additionally, these fractions reduced cyclooxygenase-2, inducible nitric oxide synthase, as well as the production of nitric oxide and prostaglandin E2. The results obtained in this study suggested that *A. scoparia* possesses potent anti-inflammatory qualities [146]. At a concentration of 50 μg/mL, the ethanolic extract of this plant reduced the expression of inflammatory genes. In 3T3-L1, RAW264.7, and 832/13 rat insulinoma cells, the extract reduced inflammatory reactions via two different signalling pathways [143]. Ahn et al [147] also reported the potential anti-inflammatory properties of the water extract from this plant. Additionally, coumarins obtained from this plant are also reported to have strong anti-inflammatory properties. In rats with adjuvant arthritis, Dou, Y. et al [149] found that scopoletin reduced synovial inflammation and bone plus cartilage destruction. It is important to mention that scoparone has potential anti-inflammatory activity; it is reported to decrease the level of cytoplasmic calcium, and can greatly relax the airway smooth muscles. In asthmatic guinea pig model, scoparone can greatly reduce inflammation in pulmonary tissues of guinea pig [152]. When combined, these findings prove that *A. scoparia* is a strong anti-inflammatory agent and that it can greatly reduce inflammation in conditions that are consistent with metabolic dysregulation. Similarly, additional research is required to demonstrate the plant’s ability to effectively reduce inflammation.

This plant is also reported to have significant antioxidant properties mainly due to its flavonoids and phenolic components, which reduce oxidative stress and can scavenge free radicals [173]. These bioactive metabolites are essential for avoiding oxidative damage to cells and tissues as well as for neutralizing dangerous reactive oxygen species. The antioxidant properties of scoparone and scopoletin from *A. scoparia* were examined by Zhu et al., compared to scoparone, the scopoletin exhibited a greater potential for radical scavenging [212]. *Artemisia* spp. include phenolic compounds, such as rosmarinic acid and caffeic acid, which are well-known for their potent antioxidant activities [177,178,213]. These compounds donate hydrogen atoms or electrons to stabilize free radicals. Flavonoids, such as quercetin and kaempferol also exhibit potent anti-oxidant activities [179]. Research has shown that the residue EOs from this plant (25–200 µg/mL) when exposed to hydrogen peroxide, have potent antioxidant and radical-scavenging properties against hydrogen peroxide and hydroxyl ions [6]. Another study focused on the ability of this plant to scavenge superoxide radicals, inhibit xanthine oxidase, and DPPH radicals. Comparing the ethyl acetate fraction of this plant to positive controls, the fraction demonstrated a high level of antioxidant activity [146]. The leaf oils of this plant (25–200 μg/mL) demonstrated strong antioxidant activity, effectively scavenging 2,2-diphenyl-1-picrylhydrazyl radicals and neutralizing hydroxyl radicals plus hydrogen peroxide. In one study, phytochemical analysis showed a high monoterpenoid content, with 31 components in mature leaves and 44 in young leaves. Major compounds were β-myrcene (24.13%) in young leaves and b-cymene (27.06%) in mature leaves. The oils demonstrated significant antioxidant and DPPH radical scavenging properties, with mature leaf oil being the most effective, followed by young leaf oil [115]. Likewise, an investigation on different *Artemisia* species found in Western Anatolia revealed that the extract of *A. scoparia* exhibited 48.51% radical scavenging activity, but the EO of the same plant showed 80.08% radical scavenging activity [173]. The extracts of this plant isolated from samples of Palestine were tested for antioxidant activity using the DPPH scavenging test. With an IC_50_ value of 21.87 ± 0.71 µg/mL, the acetone extract exhibited the strongest DPPH scavenging activity [42]. The EO of this plant have shown strong antioxidant activity in Pakistan with significant DPPH and ABTS free radical scavenging activities (IC_50_ = 285 ± 0.82 µg/mL and 295 ± 0.32 µg/mL, respectively). The primary factor influencing this activity was tocopherol derivatives, which made up 47.55% of the oil [126]. There are enough reports related to the antioxidant and free radical-scavenging activity of EOs and extracts of this plant [68,174,214], which may be linked to improved metabolic functions. While there is lack of studies of the effectiveness of *A. scoparia* extract as an anti-oxidant in Middle East countries, the other *Artemisia* species has been studied in this area [214,215].

### 9.4. Hepatoprotective Activities

Yin chen (*A. scoparia* or *A. capillaris*) is most frequently used to treat liver and gallbladder disorders, such as cholestasis and jaundice. *A. scoparia* is traditionally known for its effectiveness in treating jaundice and other liver disorders [216]. Investigations on the hepatoprotective properties of the hydro-methanolic plant extract against liver damage produced by acetaminophen were carried out by Gilani & Janbaz. Mice treated with 1 g/kg of acetaminophen died 100% with the time; however, pre-treatment with 150 mg/kg of the extract decreased the mortality rate to 20%. Acetaminophen (640 mg/kg) markedly elevated blood GOT and GPT levels in rats, suggesting hepatic injury. The extract’s hepatoprotective benefits were demonstrated by the considerable reduction of GOT and GPT enzyme levels following pre-treatment of *A. scoparia* extract [70]. Subsequent research evaluated the effect of this plant extract in diet-induced obesity mice, supplemented with 0.5% (*w*/*w*) plant extract and fed with a high-fat diet. In comparison to the high-fat diet alone, the study showed that the extract supplementation dramatically decreased fasting insulin levels and raised plasma adiponectin levels. Reduced lipid droplets were seen in the liver tissues under histological evaluation in the plant extract treated group, suggesting a decrease in hepatic lipid build up. Additionally, by raising the amount of IRS-2, IR β, phosphorylation of IRS-1, Akt1, and Akt2, and activating AMPK activity, the extract from this plant improved the hepatic insulin signalling pathways. Furthermore, upon the extract supplementation, the expression of lipid synthesis-related enzymes was downregulated, the levels of sterol regulatory element-binding protein 1c, including fatty acid synthase, and HMG-CoA reductase were significantly decreased [160]. The study by Cai et al [60] completely highlights the use of this plant in the treatment of chronic liver illnesses, especially cholestasis and its use in TCM. Yin Zhi Huang, a Chinese herbal remedy, contains *A. scoparia*, and is commonly used to treat severe and chronic hepatitis. According to a recent study by Yao et al, this herbal remedy dramatically decreased adipocyte size, adipose mass, and body weight increase in C57BL/6J mice fed with a high-fat diet. These effects were associated with an increase in mitochondrial fatty acid β-oxidation, mediated by the AMPK/ACC/CPT1A pathway and a reduction in de novo lipogenesis mediated by the AMPK/SREBP-1 pathway [153]. According to Huh et al., scoparone protected the liver against external carcinogen by inhibiting the activity of xanthine oxidase, which in turn controlled the activity of glucuronyltransferase and sulfotransferase. Rats given scoparone (35 mg/kg) showed significant inhibitions in serum ALP, AST, and ALT levels [158]. In addition to scoparone, other compounds reported in *A. scoparia* like arcapillin, quercetin, isorhamnetin, and capillarisin are also reported to have potent hepatoprotective properties [1,107]. Another study investigated the potential protective benefits of rutin, a type of flavonoid extracted from *A. scoparia*, on liver damage. Its hepatoprotective properties were confirmed when rats were pre-treated with rutin (20 mg/kg), which prevented the increase in serum levels of ALT and AST caused by paracetamol [155]. Together, there are many studies that provide a strong basis for understanding why this plant is used in folklore to treat liver diseases [2,154,159,217].

### 9.5. Other Biological Effects

The neuronal protection of this plant and its important chemical constituents is well known. Promo et al investigated the possibility of using the extract of this plant to prevent Alzheimer’s disease. Six weeks after receiving a meal containing 2% (*w*/*w*) *A. scoparia*, the hypertensive rats displayed reduced levels of amyloid β and beta-site amyloid precursor protein cleaving enzyme. But compared to the control group, the treated rats had high levels of low-density lipoprotein receptor-related protein 1. Furthermore, the plant lowered phosphorylated glycogen synthase kinase 3β and markedly decreased the levels of phosphorylated tau proteins. These results imply that this plant may assist in lowering the risk of Alzheimer’s disease [71]. Above all, scopoletin, a coumarin obtained from this plant is also well known for its pharmacological effects on protecting neurons, it is reported to be an acetylcholinesterase inhibitor [187]. Priya Kashyap et al. reported the neuroprotective potential of scopoletin and its disease-modifying potential in case of Alzheimer’s disease [188]. Additionally, the neuroprotective effects of other compounds like scoparone [189] and esculetin [195] obtained from the same plant are also well known.

The relatively broad antiviral spectrum for this plant is supported by enough reports. Utilizing a pneumonic model comprising mice and Hep-2 cells, Hua et al. extracted total flavonoids from *A. scoparia* and reported their antiviral properties. The total flavonoids decreased the ability of influenza virus to reproduce in vivo and in vitro at different doses [171]. In one study, ethanolic extract of this plant was found to exhibit anti-Hepatitis B virus activities [105]. According to Zhang Ting et al., quercetin and rutin derived from this plant demonstrated anti-SARS activities via both the traditional and alternative complement system pathways [125]. It has also been reported that several important chemicals derived from *A. scoparia* have inhibitory effects on betacoronoviruses. Collectively, there are enough available reports that support the antiviral potential of this plant and its important chemical constituents [1,170,172].

As per the available reports, *A. scoparia* has positive effects in case of diabetes and obesity related disorders. There is plenty of evidence supporting the use of this plant in traditional medicine to treat diabetes and hyperglycemia. According to a 2016 study, patients with gestational diabetes who received this plant within their diet throughout the second trimester of pregnancy experienced improvements in their insulin sensitivity, plasma glucose levels during fasting, and the level of circulating adiponectin hormone [135]. Another study proved that intake of this plant decreased the amount of cholesterol and triglycerides in the liver and increased insulin-induced phosphorylation. Furthermore, with the observed improvements in hepatic lipid accumulation in the liver, this plant increased adenosine monophosphate activated protein kinase activity and decreased the expression levels of genes linked with de novo lipogenesis [160]. According to Anik Boudreau et al., ethanolic extracts of this plant also caused antilipolytic changes in the phosphorylation of perilipin and hormone-sensitive lipase in cultured adipocytes [180,218]. Richard et al., also reported that in diet-induced obesity, an ethanol extract of *A. scoparia* showed positive effects on a number of metabolic markers [144]. The plant has been reported to reduce postprandial hyperglycemia by inhibiting alpha-amylase and alpha-glucosidase [182]. The aerial parts from this plant are reported to inhibit triglyceride accumulation that shows the importance of this plant to improve insulin resistance due to obesity [103]. By blocking lipid production and the TLR4-MyD88 pathways, the bioactive compound scopoletin derived from this plant is said to offer protection against steatosis and inflammation induced by diabetes [183]. Additionally, by inhibiting the end products production of advanced glycation and anti-glycation, scopoletin is believed to offer protection against insulin resistance and hyperglycemia induced by methylglyoxal [88]. However, it has also been shown that another compound scoparone from the same plant prevents the accumulation of triglycerides in mature adipocytes [185]. All of these reports point to this plant as a potentially beneficial dietary supplement for preventing obesity, diabetes mellitus, hyperglycemia, and hyperlipidemia.

Furthermore, available reports indicate that *A. scoparia* has promising cardiovascular benefits and may be a novel therapeutic agent for the treatment of osteoarthritis. In different countries, this plant has long been utilized to cure arthritis, according to Lu et al [219], scoparone, a coumarin derivative, which is obtained in maximum quantity during the flowering period of this plant inhibited IL-1β-induced inflammatory response in osteoarthritis chondrocytes. It has been shown that the coumarins in this plant relax blood vessels, encourage the release of prostacyclin from vascular endothelial cells, reduce hyperlipidemia, and have anticoagulant properties. In spontaneparveenously hypertensive rats, Cho et al. found that supplementing the food with an aqueous extract of this plant reduced blood pressure and had other positive effects in experimental rats [198]. According to Bao Fu et al., scoparone blocked the extracellular matrix proteins of cardiac fibroblasts generated by Angiotensin II and prevented the differentiation of cardiac fibroblasts into myofibroblasts [77]. Additionally, Yu et al [122] found that the flavonoids from this plant have antiatherogenic properties and can be helpful in curing acute myocardial ischemia.

## 10. Use of *Artemisia* Species in Poultry Feed and Implications for *A. scoparia*

Plant-derived additives have shown promising effects on poultry performance and sustainability in arid regions, much like other unconventional feed supplies like marine algae [220]. Also, using plant products plus probiotics as poultry feed can reduce heat stress and increase physiological resistance of poultry in arid areas [221]. Even though there are no specific studies about the use of *A. scoparia* as a feed additive or nutrient for different breeds of poultry, information can be obtained from studies conducted on other Artemisia species, like *Artemisia annua*, *Artemisia absinthium*, and *Artemisia capillaris*; these plant species have chemical constituents similar to those of *A. scoparia*. The supplementation of *Artemisia annua* has been documented to enrich growth performance, nutrient conversion efficacy, antioxidant characteristics, and the innate immunity of poultry as well as other farm animals. Evidence from closely related *Artemisia* species provides a preliminary basis for investigating the potential use of this plant as a poultry feed additive. Controlled in vivo feeding studies in related species have reported that low dietary inclusion rates (approximately 0.6%) were associated with improvements in body weight, antioxidant enzyme activities, and anti-inflammatory gene expression, together with reductions in oxidative stress and inflammation [222,223]. These findings suggest that certain *Artemisia* preparations may have potential as natural feed additives for supporting poultry growth and health and as possible alternatives to antibiotic growth promoters. However, these findings cannot be directly extrapolated to *A. scoparia*. Species-specific controlled feeding trials are therefore required to evaluate the safety, palatability, optimal inclusion rate, effects on growth performance and antioxidant status, and overall health effects of *A. scoparia* in poultry, particularly under arid-region production conditions. Early nutritional management can reduce heat-stress-related physiological disruption and improve production stability in broilers [224]. *Artemisia annua* supplementation has been shown to enhance the diversity of the gastrointestinal microbiota by increasing the levels of beneficial bacteria like Bacteroides and *Fecalibacterium*, which led to improved intestinal morphology as well as increase in the serum IgA, IgM, IgG, and lysozyme [225]. Studies on *A. absinthium* and *A. capillaris* also show a positive influence of the inclusion of these plants in poultry feed on the performance traits and quality of products obtained, such as increased egg productivity and a favorable profile of fatty acids, which has been attributed to the richness of its bioactive compounds [126,226,227]. Since *A. scoparia* has been reported to have almost similar chemical compositions and as mentioned above it exhibits potential biological properties [122,207], it would be reasonable to hypothesize that the inclusion of this plant in poultry diets may bring about similar biological effects, such as enhancement of oxidative status, gut microbiota modulation, and boosted immunity. However, no feeding trial has been conducted with *A. scoparia* till this date, and therefore dedicated studies need to be carried out to establish its safety, optimization of inclusion levels, and its impact on productive performance and health indices, as well as its potential to replace chemical additives used in commercial poultry nutrition. Studies conducted have proved the advantages of using phytogenic feed additives [228] and plant materials [229,230] when feeding poultry in challenging environmental conditions. Like these plants, *A. scoparia* has a great potential for usage as a locally accessible source of poultry feed in arid climates. Probiotics and prebiotics can be paired with phytogenic additives to support gut health and reduce antibiotic dependence in poultry [231]. Antioxidant supplementation combined with feed-management strategies can further improve thermotolerance under heat stress [232].

## 11. Safety, Toxicological Considerations, and Translational Limitations

This plant has been studied for its pharmacological activities, and there are different in vitro experiments and animal studies that have shown its potential hepatoprotective, anti-inflammatory, neuroprotective, and other biological activities. As per the reports related to the biological potential of plants, it is difficult to evaluate the well-established therapeutic efficacy of different plant extracts due to a lack of standardized extraction methods and inconsistent dosing regimens. Due to the differences in metabolism and bioavailability, the results obtained from the preclinical studies do not always support the therapeutic use of the plant extracts for human applications [233]. Although *A. scoparia* shows the presence of some key constituents like flavonoids, coumarins, phenolic acids, and EOs in large quantities, unfortunately, the biological mechanism of action of these bioactive constituents is not well known. The available information related to this plant shows that most of the studies have failed to purify individual compounds, and researchers have preferred to use crude extracts to explore the biological potential of this plant. In the case of crude extracts, conclusive decisions cannot be made about their efficacy because these extracts contain multiple bioactive components that may act synergistically or antagonistically [234]. It makes it more challenging to report the exact biological mechanisms of action and may hamper the reproducibility of results. More importantly, to identify the adverse effects, *A. scoparia* completely lacks detailed toxicity studies. The available literature shows hardly any study reporting human toxicity. However, a recent study by Ding et al., suggests the urgent need for examining the adverse effects of this plant [1]. Due to the limited pharmacokinetic and pharmacodynamic studies, the metabolism and absorption of the bioactive compounds from this plant remain largely unexplored. More importantly, the variability in the phytochemicals and bioactivity within the same plant highlights how important the need for standardized protocols in pharmacological evaluations is to ensure reproducibility and reliability. There is a need for future investigations for detailed mechanistic studies, systemic toxicological evaluation, extraction, and standard dosing methods to maximize the therapeutic use of this plant. A major challenge in evaluating the clinical and translational potential of *A. scoparia* is that much of the available evidence is based studies using crude extracts in cell-based in vitro models [233,234]. In addition, in vivo pharmacokinetic and toxicological data remain limited, particularly those needed to support standardized dose selection and further drug development [1]. Future studies should therefore prioritize standardized characterization of plant materials and extracts, systematic dose–response assessments, and well-designed in vivo pharmacokinetic and toxicological studies. Appropriately designed human clinical trials are also needed to generate reliable evidence regarding the safety and efficacy of *A. scoparia*. The available evidence should be interpreted in light of these limitations, as primary human clinical data remain scarce. Consequently, the safety profile of *A. scoparia* and specific toxicological endpoints have not yet been adequately characterized. Before clinical translation can be considered, further studies should establish comprehensive toxicological profiles, determine maximum tolerated doses, evaluate potential adverse effects, and investigate possible herb–drug interactions.

### Clinical Applications and Patents

Although a wide range of preclinical evidence has been reported for *A. scoparia* and its bioactive constituents, clinical evidence regarding their applications in humans remains limited. A clinical study reported potential effects of an *Artemisia*-derived preparation in gestational diabetes, including changes in insulin sensitivity and fasting plasma glucose levels [135]. However, the specific contribution of *A. scoparia* should be interpreted cautiously unless the preparation was directly attributable to this species. In TCM, *A. scoparia* has also been used as a component of formulations such as Yin Zhi Huang for conditions including neonatal jaundice and liver disorders [60,153]. However, the clinical effects of such multi-herb formulations cannot necessarily be attributed to *A. scoparia* alone. Further species-specific, well-controlled clinical studies are therefore required to establish its safety, efficacy, appropriate dosage, and clinical relevance.

From a translational perspective, research has also explored the development of extracts and bioactive constituents of *A. scoparia*, including essential oils and coumarins such as scoparone and scopoletin. Patent activity, where documented, may reflect interest in the development of extracts, formulations, or isolated constituents for potential hepatoprotective, antidiabetic, and anti-inflammatory applications. However, patent activity should not be interpreted as evidence of clinical efficacy or regulatory approval. The lack of large-scale, randomized controlled clinical trials remains an important limitation to the clinical development of *A. scoparia*-derived compounds and products. Further pharmacokinetic, toxicological, and clinical investigations are required before these compounds can be considered as validated therapeutic candidates.

## 12. Comparison with Related Species

Along with other species of medicinal importance, including *A. capillaris*, *A. annua*, and *A. absinthium*, *A. scoparia* is a member of the Artemisia genus. Although the different plant species of this genus have some similar bioactive compounds, their pharmacological effects and chemical compositions are different. The comparative study by Trifan et al., related to the phytochemical profiles of the five Artemisia species, showed that *A. vulgaris* represented the group of phytochemicals that were separate from other species, whereas the phytochemical composition of *A. annua* was similar to *A. absinthium*, and *A. pontica* was similar to *A. austriaca* [235]. Among the species of medicinal importance, *A. scoparia* is rich in EOs, flavonoids, and coumarins that are responsible for its potential anti-inflammatory, hepatoprotective, and other biological effects, whereas *A. annua* is mainly recognized for its artemisinin content, a strong antimalarial bioactive compound that is currently used to treat malaria patients [236]. Similarly, *A. absinthium* is unique because it shows the presence of thujone, a monoterpene ketone, in very high concentrations. This bioactive compound, thujone, present in *A. absinthium* is mainly responsible for its potential pharmacological properties [237]. Although *A. capillaris* and *A. scoparia* are reported to have some chemical similarity, the EOs of both the plants show the presence of β-caryophyllene. However, different phytochemical profiles are highlighted by the presence of some bioactive compounds, such as 1,8-cineole and camphor in *A. scoparia*, compared to capillene and β-pinene in *A. capillaris*. *A. capillaris* has a notably higher amount of capillene, a chemical that is less common in *A. scoparia*. The biological activities of both the plants are different as a result of differences in chemical compositions [7]. According to a study by Farzaneh et al., the essential oils of *A. aucheri*, *A. sieberi*, and *A. scoparia* are found to be rich in linalool (44.1%), β-thujone (19.8%), and alpha-thujone (81.7%), respectively. When compared to *A. scoparia*, the EOs of *A. aucheri* and *A. sieberi* have greater antifungal action against soil-borne pathogens [127]. 

A study about the chemical composition and bioactivity of five Artemisia species showed the major polar compound present in *A. scoparia* to be casticin (8.23%), while in the case of *A. dracunculus*, the major compound is reported to be chrysosplenol D (9.94%). In the same study, the major non-polar compound in *A. scoparia* is reported to be capillene (44.61%), while in the case of *A. annua*, *A. dracunculus*, and *A. santonica*, the major non-polar compounds are artemisia ketone (32.81%), estragole (56.20%), and cis-chrysanthenol (21.57%), respectively. Compared with the other four *Artemisia* species evaluated in the cited study, *A. scoparia* exhibited the highest antimicrobial activity against most of the bacterial and fungal strains tested [238]. One interesting study from Saudi Arabia indicated that the EOs obtained from three different *Artemisia* species (*scoparia*, *sieberi*, and *absinthium*) contained four bioactive compounds in common. The main compounds found in the case of *A. sieberi* and *A. absinthium* were camphor and cis-davanone. In contrast, EOs of *A. scoparia* showed the presence of 2-undecanone and 2-nonanone as the main compounds; these compounds represented 80% of the total EO content [116]. In the same study, in comparison to *A. absinthium*, the EOs of *A. sieberi* and *A. scoparia* exhibited the most notable activity against both Gram-positive and Gram-negative bacteria. *A. scoparia* has a unique phytochemical composition and a wider range of biological activities, even though it consists of some chemical compounds that are also found in some other species of the same genus [239,240]. These results emphasize the need for more targeted research and support its potential as a valuable medicinal plant.

## 13. Potential for Sustainable Utilization

In case of *A. scoparia*, the increase in biomass is linked to decrease in community density and suitable seed germination [241]. This plant can be used as a grazing crop, owing to its fast growth, higher biomass production. It is also a source of organic herbicide owing to the phytotoxic effect of *Artemisia* oil. The oil from this plant is reported to interfere with cellular respiration and loss of chlorophyll content in weeds [113], phenyldiacetylene is reported to be the major constituent of *A. scoparia* oil [50]. This plant is not a fodder crop due to its bitter taste [66], but it has proven ability to provide large number of useful metabolites of medicinal importance. The plant generally tends to reduce its growth for salt tolerance [242], and is one of the limited options for rangeland development. Since this plant is an annual or short living perennial plant and is ought to grow in rainy season and survive until it reaches the threshold of draught tolerance. Biochar-based poultry strategies illustrate how circular bioeconomy tools can complement native-plant-based approaches in sustainable dryland agriculture [243]. The extreme draught tolerance and the survival of this plant in the harsh desert conditions with diverse range of phytochemicals makes this plant a potent crop of pharmaceutical importance. Since most of the agricultural activities in Arabian Peninsula and Middle East countries including Kuwait are based on fodder generation for livestock, this plant makes it a best companion plant to reduce the pests. Besides pest reduction, the oil from this plant shows activity against stored product insects and thus plays a vital role in protecting organic food [8]. A recent study reports that *A. scoparia* straw has a variety of applications in nanocomposites and the ability to extract cellulosic nanomaterial [244]. In Kuwait, the use of native plants has been reported as a sustainable strategy for improving feed resources in poultry systems [245]. Since no poultry feeding trails have been performed in case of *A. scoparia*, more research is needed to determine its safety levels, and effects on poultry health. In Kuwait and neighbouring countries, the best method for increasing feed supplies in poultry systems is the use of native plants that have grown to arid environmental conditions. Water-scarcity solutions such as solar water disinfection highlight the need for low-resource, climate-adapted strategies in arid-region food systems [246]. Also, this plant is recently getting attention and has a greater potential for use as a commercial crop. The available reports about the chemical composition and biological potential of this plant highlights its significance in both traditional and modern medicinal uses, making it important for further scientific investigation.

## 14. Knowledge Gaps and Future Research Needs

*A. scoparia*, an important medicinal plant has a variety of chemical constituents and bioactivities, nonetheless, there is not enough research to fully understand this plant as a promising and complete therapeutic agent, despite the fact that we have reviewed plenty of reports on its biological potential and metabolic benefits. The broad spectrum of bioactivities of this plant when treated against various cell lines, pathogenic microorganisms, and disease models further supports the need to study the plant in humans. Additionally, the fact that this plant is widely used in traditional medicine worldwide [48,49], points to promising safety and toxicity research. In order to evaluate the results of various extracts and to guide the pharmacokinetic evaluation of possible therapeutic extracts from this plant, rigorous characterization using standardized procedures is essential. To create the foundation for pre-clinical research, more animal studies would be beneficial. The different biological activities and chemical makeup of this plant may also be used as a resource to correlate the bioactivity of constituent chemicals. It is necessary to examine the antioxidant and hepatoprotective properties in relation to metabolic disease. Despite of well-known biological activities of this plant, there are still a number of unexplored study fields. The potential of this plant for cancer treatment is one encouraging area, that needs further research. Although thorough mechanistic research and clinical assessments are absent, recent studies indicate that coumarins and flavonoids present in this plant could impact important pathways of cancer [2]. More research is required to determine whether this plant can help people with diabetes, the need is to examine its effects on metabolic health measures like glycemic control, insulin sensitivity, or cardiovascular risk factors. Furthermore, there is growing interest in the potential of essential oils extracted from this plant as natural antimicrobial agents for infections that are resistant to standard drugs, in this area large-scale pharmacological validations are required. The mechanism of the biological action of this plant is still only partially understood; further clarification of the mechanistic details and implications of *A. scoparia* activities is necessary to fully fulfill its potential as a valid and promising therapy. 

**Figure 4:**
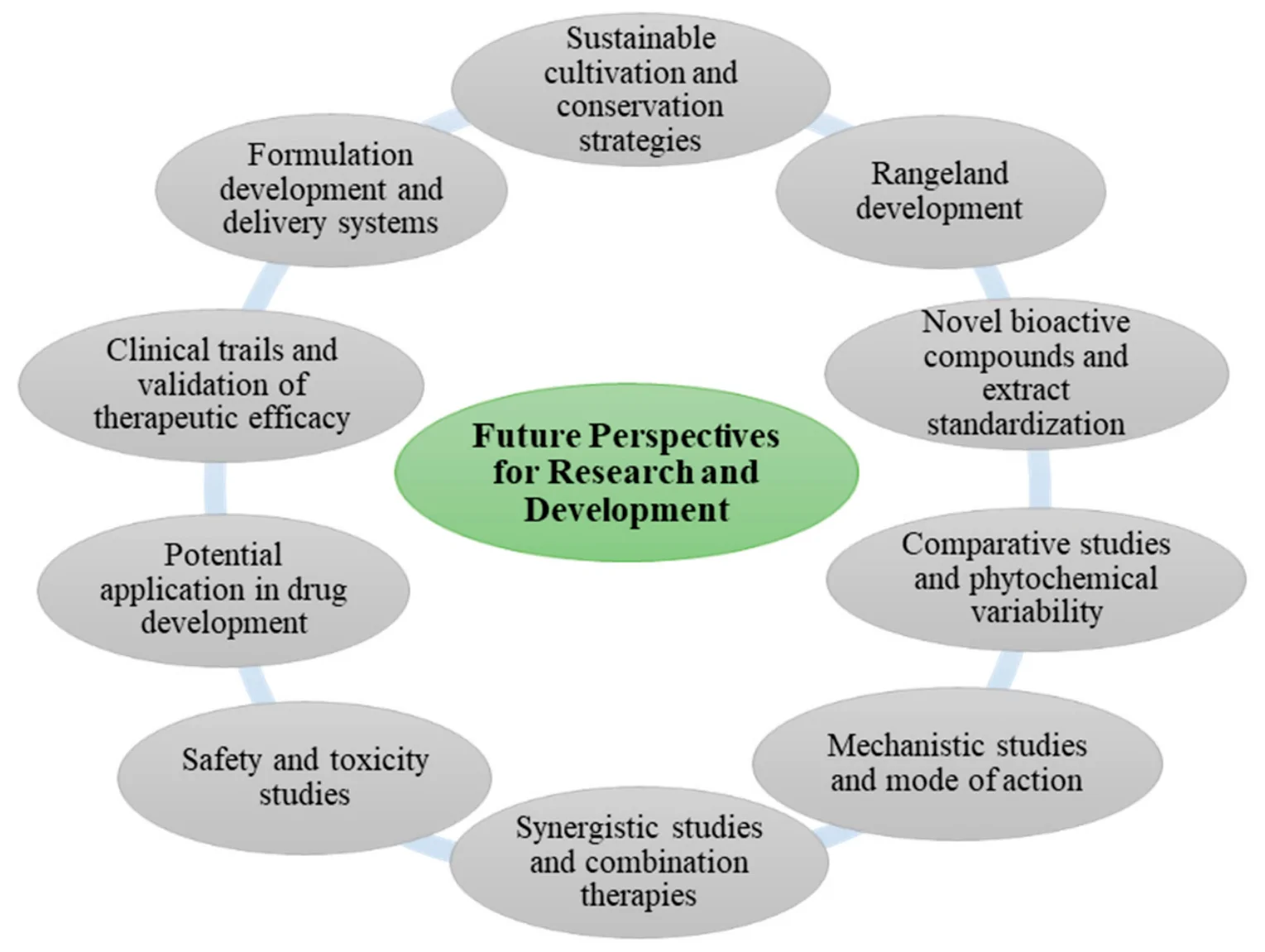
Future perspectives for research and development on *A. scoparia*.

The promising medicinal properties of *A. scoparia* are well known; still, using it in clinical settings presents several difficulties. Strict approval procedures required for plant-based medications, which include drug safety, their effectiveness, and toxicity evidence before the drug is being evaluated for clinical use, are examples of some important regulatory barriers [233]. As per the reports, the majority of research related to the medicinal use of *A. scoparia* is still in the preclinical stage, and there are hardly any clinical studies for the bioactive compounds derived from this plant. It is suggested that in the case of this plant, randomized controlled clinical trials must be conducted to confirm the optimal dosage, toxicity levels, and potential drug interactions. Additionally, the bioavailability of important bioactive compounds from this plant, like flavonoids and coumarins, must also be addressed to increase the therapeutic efficacy of this plant. Currently, formulation strategies are very important for overcoming these challenges. Available encapsulation approaches [247] can be used to improve the targeted administration and regulated release of the bioactive extracts from this plant, which can increase its therapeutic benefits while minimizing side effects. The notable bioactivities draw attention to potential avenues for developing novel drugs. However, more thorough investigations are needed to prove that this plant is a reliable therapeutic agent. (Figure 4) highlights future perspectives for research and development on *A. scoparia*.

In order to improve desert ecosystems and conserve biodiversity in Kuwait and its neighboring countries, restoration approaches and rangeland management initiatives may benefit from an understanding of the germination strategies of this plant and other native plants [18] linked to drought stress and the identification of favorable conditions throughout the germination process [248]. In addition to this, the allelopathic interaction of *Artemisia* species is well known. Influence of nurse shrubs, maintaining diversity in *Artemisia* species and interactions with other plants with respect to *A. scoparia* is yet to be studied [249]. Many *Artemisia* species are studied for their endophytic communities [250,251]. Even *A. scoparia* is reported to be studied for endophytes from Asia [252,253], but the data from the Arabian Peninsula and Middle East countries are missing. The salinity stress trait of this plant has been studied in vitro [36], but not in the desert ecosystems, including Kuwait. The antioxidants are proven to be expressed in water stress conditions [254], and environmental abiotic restrictions [255], but there is a lack of data from arid regions like Kuwait. The symbiotic relations of plant growth promoting microbes with species like *A. annua* are studied [256], unfortunately, there are no references available on *A. scoparia*. The interactions between mycorrhiza and fungal endophytes that contribute to drought stress tolerance, as well as the possible mechanisms by which arbuscular mycorrhizal fungi may promote drought tolerance in this plant, require more investigation [38]. 

Despite promising indications based on related Artemisia species, there is a lack of species-specific data regarding safety, bioavailability, and efficacy of *A. scoparia* in poultry feed. It is recommended that future studies focus more on feeding trials in poultry to assess the toxicological properties, optimal inclusion rates, and effects on poultry performance and health.

Also, the studies related to the forage potential of this plant are very less, little efforts are seen in the conservation efforts of this plant. *A. scoparia*, however has high dominance in seedling germination as compared to other species of *Artemesia*, but this plant is not studied as a candidate in conservational efforts for arid rangeland development.

## 15. Conclusions

*A. scoparia* is an ecologically adaptable species that can persist under arid and semi-arid conditions across parts of the Middle East, the Arabian Peninsula, and Asia. Its long history of traditional medicinal use, together with its diverse phytochemical profile and reported biological activities, highlights its potential for further investigation in multidisciplinary biomedical, ecological, and agricultural contexts. Its drought adaptability and reported phytotoxic properties may provide opportunities for applications in arid-land ecological restoration and bioherbicide development; however, these applications require further experimental validation under relevant field conditions. Similarly, evidence from related *Artemisia* species provides a preliminary rationale for investigating the potential use of *A. scoparia* as a poultry feed additive in dry regions, but this application remains hypothetical because species-specific feeding trials and safety assessments are currently lacking.

To clarify the clinical and translational potential of *A. scoparia*, future research should prioritize standardized phytochemical characterization, well-defined toxicological assessment, pharmacokinetic studies, dose–response evaluation, and appropriately designed human clinical trials. Further research should also establish specific safety endpoints and clarify the molecular mechanisms underlying its reported biological activities. Such evidence will be essential for determining whether the pharmacological, ecological, and agricultural potential of *A. scoparia* can be translated into safe, evidence-based applications in medicine and sustainable land management.

## Figures and Tables

**Table 1:** List of the countries where *A. scoparia* is native and introduced [19].

Native to
Pakistan, Egypt, Saudi Arabia, Iran, Turkey, Kuwait, Germany, Poland, Uzbekistan, India, Hungary, China, Switzerland, Russia, Finland, Iraq, Romania, Myanmar, Afghanistan, Switzerland, Netherlands, Austria, Turkmenistan, Japan, Kazakhstan, North Caucasus, Gulf States, Czechoslovakia, Mongolia, Tibet, Albania, Bulgaria, Ukraine, Yugoslavia, Greece, Italy, West Himalaya, Tajikistan, Kyrgyzstan
Introduced into
Belgium, Cambodia, Thailand, Belarus, Malaya, Great Britain, Palestine, Baltic States, Borneo, Vietnam, Taiwan, Sinai, Jawa, Maluku, Sumatera, Korea, Maryland, Laos, Lebanon-Syria, Philippines

**Table 2:** Traditional uses of *A. scoparia* in different countries.

Country	Used to Cure	Plant Parts Used	References
Pakistan	Cancer, diabetes, hepatitis	Root, leaves	[48,53]
	Hepatitis, jaundice, improve digestion	Leaves	[54]
	Parasitic infections, improve digestion, hair, snake or scorpion venom, malaria, hepatitis, jaundice, used as purgative	Whole plant, young shoots, flowers, leaves	[44,55]
	Fever, snake or scorpion venom, burns, skin/hair, respiratory ailments, gastric problems, malaria, used as vermifuge, depurative, and purgative	Twigs	[11,56]
	Parasitic infections, fever, cardiovascular problems, cough	Whole plant	[57,58]
China	High blood pressure, diabetes, jaundice, improve digestion, respiratory problems, typhoid, clears phlegm	Root, flowers, leaves	[59]
	Jaundice, skin, fever, malaria, osteoarthropathy, gall bladder inflammation, parasitic infections, snake or scorpion venom, hepatitis, liver diseases, sores, itching, asthma, gastritis, phlegm detoxification	Aerial parts, whole plant	[1,60]
India	Respiratory ailments, diabetes, wounds, cough, cold, used as depurative and colic	Leaves	[61]
	Liver infections, digestion, joint pain, parasitic infections	Leaves	[51]
	Dental problems	Leaves, seeds	[62]
Saudi Arabia	Hepatitis, gall bladder inflammation, digestion, fever, jaundice used as an antiseptic, diuretic, purgative, antibacterial, anticholesterolemic, cholagogue, and vasodilator	Whole plant	[63]
	Jaundice, liver disorder	Aerial parts	[49]
Iran	Flatulency in children, improve digestion, skin, joint pain and rheumatism, hydrocele	Flower head, leaves	[64,65]
	Hypoglycemic, hypolipidimic, used as anti-inflammatory	Aerial parts	[66]
Afghanistan	Urinary tract inflammation, skin infection, used as antirheumatic, antigout, and diuretic	Whole plant	[67]
Mongolia	Parasitic infections, stomachache, dental ailments, used as antiseptic and anti-inflammatory	Whole plant	[52]

**Table 3:** Plant extracts, essential oils, pure compounds, and nanoparticles derived from *A. scoparia* responsible for various biological activities.

Antimicrobial	Anticancer
Essential oils [7,116,126,127] Oxygenated monoterpenes camphor, 1,8-cineole and the sesquiterpene hydrocarbon β-caryophyllene [7] α- and β-thujones [138,139] 1,8-cineole [140] Ethyl acetate, aqueous, acetone, ethanol, and n-hexane extracts [68] Methanolic extract [141] Ethyl acetate, diethyl ether, and hexane extracts [142] Hexane and acetone extracts [42]	Zinc oxide nanoparticles synthesized using *A. scoparia* extract [128] Methanolic extracts [68,69,129] Scoparone [121] Scopoletin [130,131,132] Silver nanoparticles synthesized using *A. scoparia* extract [133] Chitosan and myristic acid nanogel-encapsulated *A. scoparia* extract [134] Quercetin [135,136,137] Combination of quercetin and alantolactone [35]
**Hepatoprotective activities**	**Anti-inflammatory**
*A. scoparia* present in Yin Zhi Huang, a traditional Chinese herbal medicine [153] Crude extract from aerial parts [70,154] Rutin (flavonoid) [155,156,157] Scoparone [78,158,159] 80% ethanol extract [160] Hydro-methanolic extract [70] *Artemisia scoparia* Herba (Traditional Chinese Medicine) [60] Scopoletin [161,162] Esculetin [163,164] Flavanols [165,166,167] Flavanones [168] Phenolic acids [169]	Ethanolic extract [143,144,145] Hydromethanolic extract [12] 3,5-dicaffeoyl-epi-quinic acid isolated from *A. scoparia* butanol fraction [102] Ethylacetate fraction [146] 80% aqueous ethanolic extract of the aerial part [103] Water extract [147] Butanol-extracted fraction of *A. scoparia* and 3,5-dicaffeoyl-epi-quinic acid [148] Scopoletin [149,150,151] Scoparone [81,152] Crude extract, aqueous fraction and total flavonoid contents [68] Flavonoids [40]
**Antiviral**	**Antioxidant**
Total flavone content [1,170,171] 90% ethanolic extracts [105] Cirsimaritin [172] Rutin and quercetin [125]	Essential oils [6,106,126,173] Acetone, ethanolic, and aqueous extracts [42] Methanolic extract [173,174,175] Scoparone [176] Rosmarinic acid and caffeic acid (Phenolic compounds) [177,178] Quercetin and Kaempferol (Flavonoids) [179] Ethyl acetate fraction [146]
**Antiobesity and Antidiabetic**	**Neuroprotection**
Acetone and aqueous extracts [42] Ethanol extracts of *A. scoparia* [42,103,135,145,160,180,181] Scopoletin [88,182,183,184] Scoparone [185] Crude extract [135] Methylene chloride and methanol extract [186]	Aqueous extract of aerial parts [71] Scopoletin [71,187,188,189,190,191,192,193] Scoparone [189,194] Esculetin [195,196] Phenolic acids [102,148,197]
**Cardiovascular effects**	
Scoparone [75,194] Hot water extracts of aerial parts [198] Scopoletin [199,200] Esculetin [201,202] Rutin [203] Phenolic acids [204,205] Flavonoids [122,123,124]	

## Data Availability

No new data were created or analyzed in this study. Data sharing is not applicable to this article.

## Abbreviations

EOs	Essential Oils
USDA	United States Department of Agriculture
CAM	Crassulacean acid metabolism
TCM	Traditional Chinese Medicine
MIC	Minimum Inhibitory Concentration
ER-a	Estrogen Receptor-a
STAT3	Signal Transducer and Activator of Transcription
DEQA	3,5-dicaffeoyl-epi-quinic acid
